# When the firm prevents the crash: Avoiding market collapse with partial control

**DOI:** 10.1371/journal.pone.0181925

**Published:** 2017-08-23

**Authors:** Asaf Levi, Juan Sabuco, Miguel A. F. Sanjuán

**Affiliations:** 1 Nonlinear Dynamics, Chaos and Complex Systems Group. Departamento de Física, Universidad Rey Juan Carlos, Tulipán s/n, 28933 Móstoles, Madrid, Spain; 2 Institute for Physical Science and Technology, University of Maryland, College Park, Maryland 20742, United States of America; University of Rijeka, CROATIA

## Abstract

Market collapse is one of the most dramatic events in economics. Such a catastrophic event can emerge from the nonlinear interactions between the economic agents at the micro level of the economy. Transient chaos might be a good description of how a collapsing market behaves. In this work, we apply a new control method, the partial control method, with the goal of avoiding this disastrous event. Contrary to common control methods that try to influence the system from the outside, here the market is controlled from the bottom up by one of the most basic components of the market—the firm. This is the first time that the partial control method is applied on a strictly economical system in which we also introduce external disturbances. We show how the firm is capable of controlling the system avoiding the collapse by only adjusting the selling price of the product or the quantity of production in accordance to the market circumstances. Additionally, we demonstrate how a firm with a large market share is capable of influencing the demand achieving price stability across the retail and wholesale markets. Furthermore, we prove that the control applied in both cases is much smaller than the external disturbances.

## Introduction

Economic dynamics constitute an important research field in economics. Many models have been developed to explain the motion of economic variables such as the price, the demand or the GDP, giving rise to different dynamical behaviors, like periodic orbits, strange attractors and equilibrium states. But, sometimes, extreme events lead to a market collapse. Economists agree that market collapses are characterized by an abrupt fluctuation or a chain of fluctuations that decreases the value of some economical variable dramatically.

**Fig 1:**
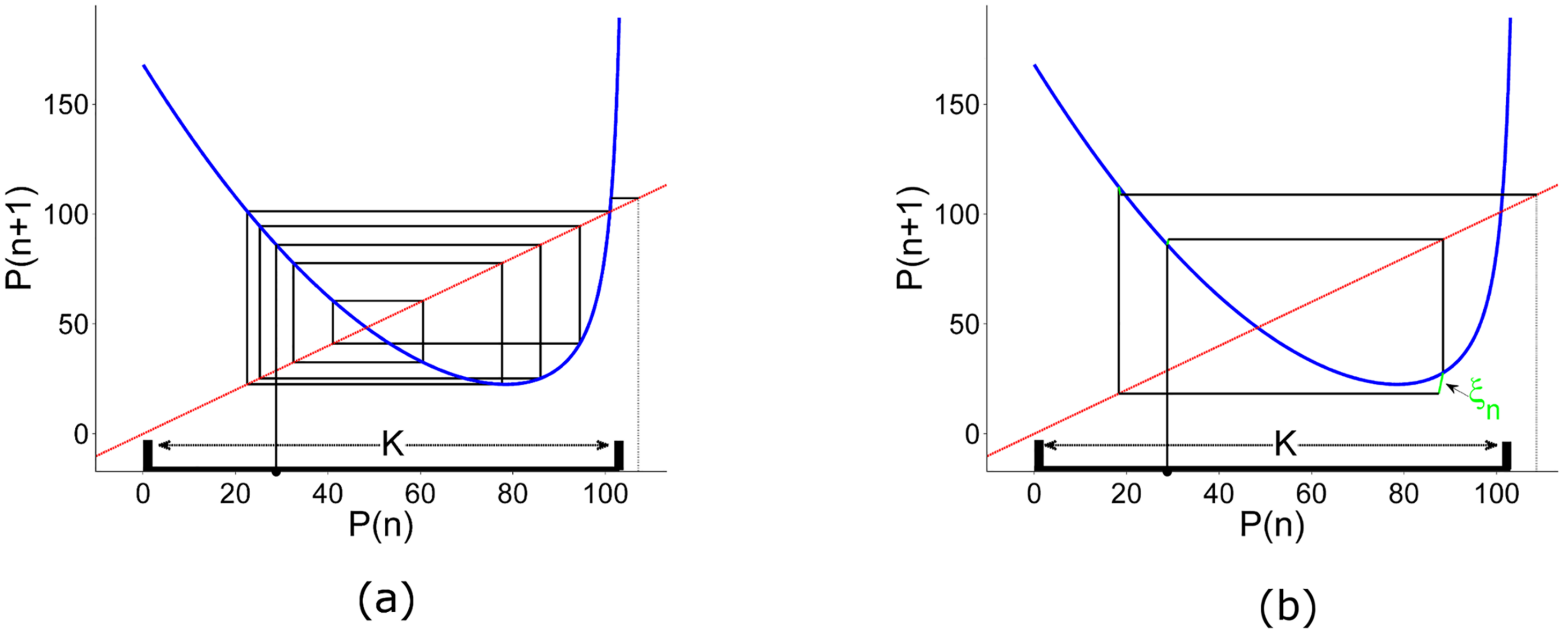
Cobweb plots of the price map. The blue line represents the price map, the diagonal red dashed line represents *P*_*n*+1_ = *P*_*n*_. The initial condition in both panels is *P*(1) = 28.8. When no disturbances nor control are present in the system, after a few iterations the trajectory escapes (black dotted line) from the chaotic saddle (region K) as shown in panel (a). When a disturbance, *ξ*_0_ = 10.0 is present in the system the trajectory escapes from the chaotic saddle even faster as shown in panel (b). The green lines represent the amount of disturbance introduced into the system at each time step. Notice that each line has a different length because it is defined by a uniform distribution function bounded by *ξ*_0_ = 10.0. The black thick line over the horizontal represents the region K which is the region where we want to sustain the dynamics.

In this work, we consider a particular dynamical behavior called *transient chaos*. This phenomenon can be found in many systems such as a thermal pulse combustor [1] a periodically driven CO2 laser [2], a voltage collapse [3] or a three-species food chain ecological model [4]. In economics, transient chaos can be found in many systems as well, such as speculative markets models [5–7], a business cycle model [8] and a duopoly model [9]. The topological structure behind this behavior is the presence of a chaotic saddle in the phase space. This topological object arises when a chaotic attractor collides with its own basin boundary producing a transient chaotic behavior before eventually escaping towards an external attractor [10] as shown in panel (a) in Fig 1.

**Fig 2:**
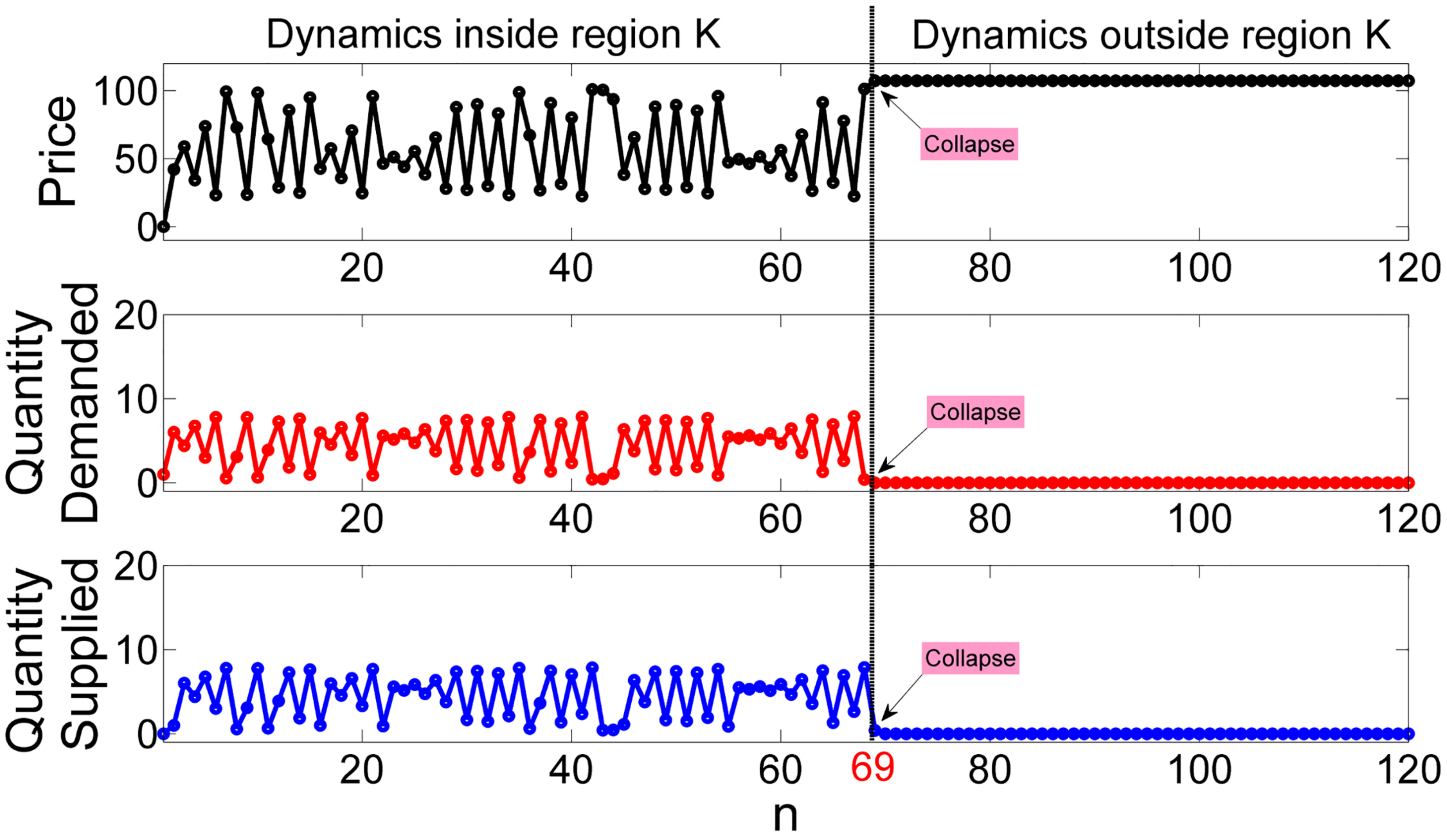
Time series of the quantity demanded, the quantity supplied and the price inside and outside region K. When the trajectory is inside region K, it follows a chaotic dynamics governed by the maps of the price, the demand and the supply in Eqs (11–13). After some critical value is crossed, here in time step 69, the system escapes from region K and the dynamics changes: the price stays fix at some high level, *P*_*n*+1_ = *P*_*n*_ in which there is no demand *D*_*n*+1_ = 0. Owing to the absence of demand the firm losses the incentives to produce, *S*_*n*+1_ = 0 and the market suddenly collapses.

A collapsing market behaves similarly to a transient chaotic system, where the fluctuations of the price, the demand or the supply are erratic but bounded, until they reach to some critical value after which the whole system collapses as shown in Fig 2. We have chosen the supply based on demand (SBOD) model to study transient chaos in the economy [11]. This model is based on the classic cobweb model [12, 13], with the difference that the firm tries to adjust the production in accordance with the expected demand, instead of the expected price. In some way, this model is an iterative deterministic version of the Newsvendor problem [14]. The main differences between these two models are that in the SBOD model we assume a deterministic demand function that depends on the selling price instead of some random demand function. Additionally, we assume that the process of stocking is an iterative process. The interest of this model relies on the simple explanation of how small firms prepare their inventory for the coming sales season computing the expected demand using a simple model and some past sales data. This model produces the following dynamics: equilibria points, periodic orbits and chaotic behavior which for some parameter values becomes transient. In this situation the trajectories of the price, the demand or the supply are chaotic some time until they eventually collapse. In the context of our model, we mean by market collapse a market state characterized by high prices in which the firm loses the incentives to supply due to the very low or even zero expected demand. When the supply and demand vanish the trade becomes impossible and the market collapses. This behavior is shown in Fig 2.

When the system falls in the market collapse state, the question that naturally arises is the possibility of avoiding it, maintaining the system in the transient regime. Three problems arise when trying to control any economical system. The first one is the prediction problem. How do we know beforehand that the market is close to a collapse? A lot of research has been done to answer this question. A few interesting works focused on the stock markets can be found in [15–20]. The second one is where to apply the control. In many models the control is applied on some parameter that is not trackable or can only be influenced theoretically. The third problem is that all real economies are affected by certain external disturbances, producing large deviations in a nonlinear deterministic system as shown in panel (b) in Fig 1. In fact, many control methods that are effective without disturbances, can fail when the disturbances are present [21]. We have chosen this specific model because it has some parameters that can be easily controlled by the firm. For example the selling price of the product can be easily adjusted by the firm, changing the firm’s gross margin at each time step.

In recent years, a novel control method called partial control has appeared in the literature [22–25]. This control method is applied in situations where transient chaos is present and the system is subjected to external disturbances as shown in Fig 1. The main results of this paper are that the firm can successfully control the trajectories of the price by only changing the gross margin at each time step preventing a market collapse. It can also rationalize the quantity supplied with the same purpose. Moreover, we show that firms with market power can influence the demand in the retailer or wholesaler markets, generating market stability in the long run. Furthermore, we prove that the amount of control needed in those cases is even smaller than the disturbance.

The structure of the paper is as follows. Section 2 is devoted to the description of the supply based on demand model. The main ideas of the partial control method are described in Section 3. In Section 5 and 6 the safe sets are computed for the price and the quantity demanded in the naive supplier case, in order to produce controlled trajectories. In Section 7, we have computed the safe sets of the quantity supplied in the cautious and optimistic supplier case, generating controlled trajectories. Finally, some conclusions are drawn in Section 8.

## Description of the supply based on demand model

We use the supply based on demand model proposed by Levi et al [11]. This model describes the price-quantity dynamics in a market where the consumer obeys the demand law and the firm prices its products by only adding its gross margin to a quadratic average total cost function. The main assumptions done in this model are that the firm is a price maker, implying that it is the only one who sets and adjusts the price in light of circumstances, while its main goals are to sell all the produced products and to satisfy the overall demand. The general structure of the model is as follow,

$$\begin{array}{c} D_{n+1}=a-bP_{n+1}, \end{array}$$ (1)

$$\begin{array}{c} S_{n+1}=D_{n+1}^{Exp}, \end{array}$$ (2)

$$\begin{array}{c} P_{n+1}=\frac{ATC}{1-M}. \end{array}$$ (3)

**Fig 3:**
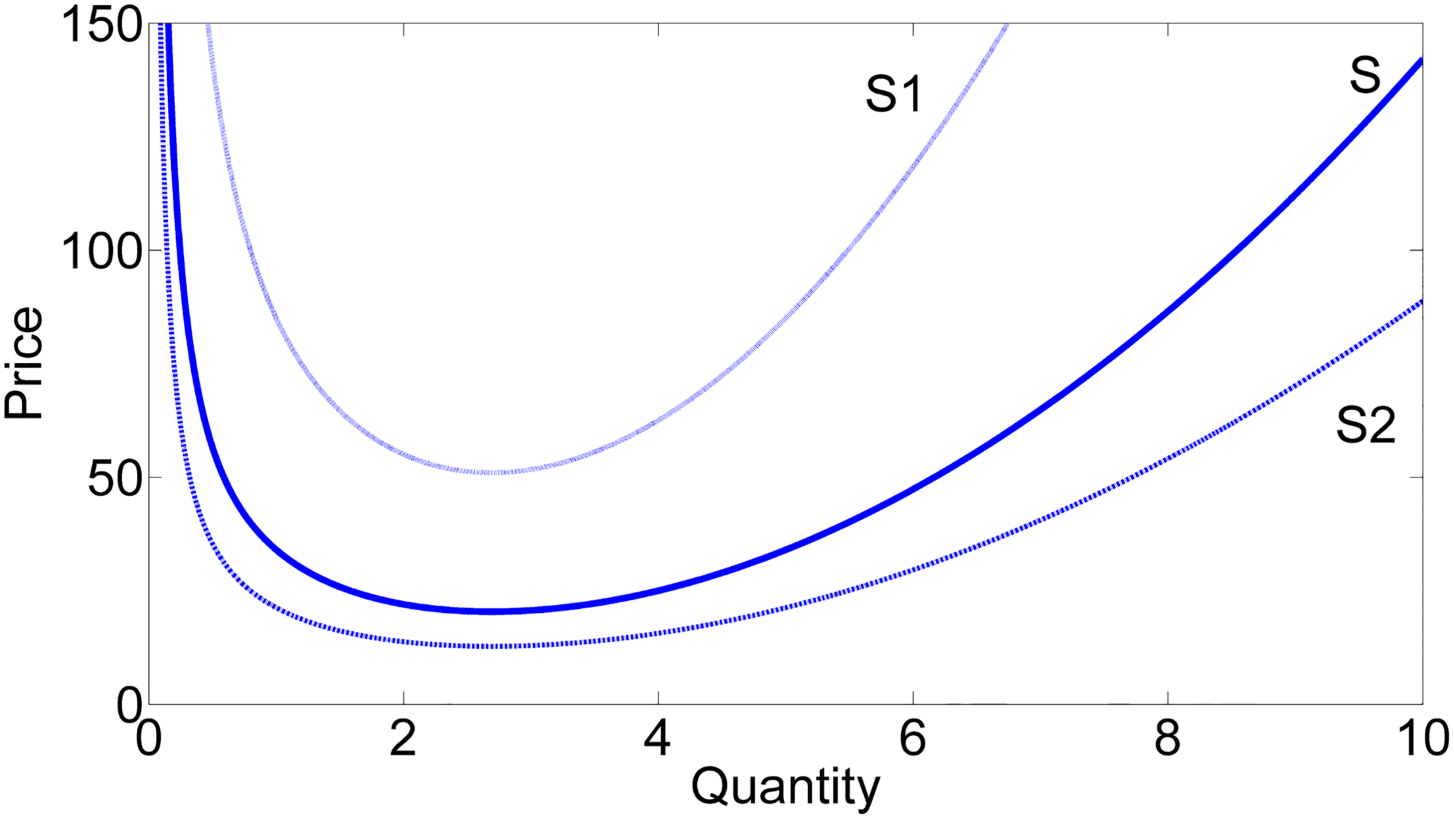
The price-quantity function. We have used the following function $P=\frac{1}{1-M}\cdot \left(\frac{F_{c}}{Q}+v-vQ+Q^{2}\right)$, to relate the price of the product with the quantity supplied, where *P* is the selling price of the product (cost + profits) and *Q* is the quantity of production. The average fix cost function is $\frac{F_{c}}{Q}$ where *F*_*c*_ is a positive constant and the average variable cost function is *v* − *vQ*+*Q*^2^, where *v* is positive constant. The parameters are fixed as: *F*_*c*_ = 10 and *v* = 4. The supply curves *S* (solid line), *S*1 (dot line) and *S*2 (dash-dot line), correspond to the gross margin *M* = 0.5, *M* = 0.8, *M* = 0.2 respectively. When the firm increases the gross margin *M*, the price increases and when the firm reduces the gross margin *M* the price decreases.

The quantities demanded and supplied, *D*_*n*+1_, and *S*_*n*+1_, and the price, *P*_*n*+1_ are assumed to be discrete functions of time. The parameters *a* and *b* are positive constants *a*, *b* ≥ 0. The firm expected demand is $D_{n+1}^{Exp}$, and *M* is the gross margin added by the firm to obtain profits, where 0 ≤ *M* < 1. The average total cost function *ATC* of the firm will adopt a U-shape, when diminishing returns are present in the production process and the firm has variable costs. Applying this idea, when the firm increases the amount of production the average total cost of every unit of production decreases until it reaches some critical point from which every additional produced product will increase the unit average total cost. In the decreasing side of the curve, the firm enjoys of scale economies, that is, decreasing returns to scale. After crossing this point every additional product produced increments the average total cost of the firm which implies a diminishing returns to scale [26]. The U-shape of the ATC function as shown in Fig 3 captures this idea. In our case, the quantity of production is the same as the quantity of supply, *S*_*n*+1_, or the expected demand estimated by the firm, as shown in Eqs (2) and (4),

$$\begin{array}{c} ATC=\frac{F_{c}}{S_{n+1}}+v-vS_{n+1}+{\left(S_{n+1}\right)}^{2}. \end{array}$$ (4)

We assume that *v* and *F*_*c*_, are positive constants. In order to obtain profits, the supplier adds over the average total cost of the product some quantity using the gross margin operator shown in Eq (3). When *M* increases, the ATC function moves upwards, what leads to higher selling prices and when it decreases the ATC moves downwards what leads to a cheaper products as shown in Fig 3. We assume that the selling price is in fact the market price.

The firm makes the decision of what quantity to supply, *S*_*n*+1_, before it knows the reaction of the market to the price it fixes. The firm makes this decision based on the quantity it expects the market will demand, $D_{n+1}^{Exp}$, in the future. The firm does not know anything about the demand function. The only available information it has, is the quantity demanded at the price in which its products were sold in the last sales season. It is important to notice that the quantity demanded from the firm perspective is the sum of the total units sold and the total units out of stock (stock rupture). The firm quantifies its success after each sales season using a very simple model—it divides the quantity demanded by the quantity supplied at time *n* as shown in Eq (5). We called it the signal of success S,

$$\begin{array}{c} S=\frac{D_{n}}{S_{n}}. \end{array}$$ (5)

According to the signal of success, the firm makes the decision of how many products to produce and supply in the next sales season. We assume an ordinary goods market in which, when the price increases, the consumption decreases and vice versa. For simplicity, we assume a linear demand curve with negative slope as shown in Eq (1).

The model describes just one firm, and it does not take into account its financial constraints. Furthermore, the firm does not try to maximize its profits nor accumulate stock. The model works as follows. In the first step the firm supplies a certain amount of products to the market to get some feeling about the demand (seed). Then, it observes the amount of products that were demanded at this specific selling price. According to this quantity the firm decides how many products to produce for the next sales season using the signal of success *S* shown in Eq (5). The firm uses this signal to estimate the expected demand in the next period. The second step is the pricing process. The firm uses its ATC function to compute the products average total cost. After obtaining the cost per unit, it adds profits over the cost using the gross margin operator. Finally, it introduces the products with their new price into the market, it waits some time until it sees how many products have been sold and then it repeats all the process again at every time step. This model aims to explore the global dynamics of the market as a result of this simple behavior of the firm.

In [11] the authors focused only on two supplier types, the naive supplier and the cautious and optimistic supplier. In this work, we will show that the partial control method can be successfully used in both cases.

## Application of the partial control method

The partial control method has been successfully applied to several paradigmatic dynamical systems, such as the Heńon map, the tent map [27], the time-2*π* map associated to the Duffing oscillator [10, 24, 25] and in the 3D Lorenz map [28]. In particular, when we consider the dynamics after a boundary crisis, the system possesses a transient chaotic behavior in a bounded region in phase space, previous to a situation in which the trajectory escapes towards an attractor outside this region. When the dynamics is affected by noise, somehow it might help the trajectory to escape from the region earlier as shown in Fig 1. The goal of the partial control is to apply a control in order to avoid the escape of the trajectory from this region K, and what is surprising is that the amount of control we need is smaller than the external disturbance acting on the dynamical system. To implement this method, we need a map and to define a region K in phase space, where we want to sustain the dynamics. The complete dynamics in presence of an external disturbance *ξ*_*n*_ and after the application of a control *u*_*n*_ is described by the iterative equation *k*_*n*+1_ = *f*(*k*_*n*_) + *ξ*_*n*_ + *u*_*n*_. The only assumption we consider on the disturbances and control is that are bounded, that is, |*ξ*_*n*_| ≤ *ξ*_0_ and |*u*_*n*_| ≤ *u*_0_, and when this happens we say that we have admissible disturbances and controls. A point *k* ∈ *K* is considered safe, if the next iteration of this point f(k) under the action of the map and affected by the external disturbance can be put again on K once a control |*u*_*n*_| ≤ *u*_0_ < *ξ*_0_ is applied. We can say that under the previous considerations, a safe point is partially controlled and consequently remains in K with an applied control smaller than the disturbance. The set of all safe points in K is called the safe set. There is an algorithm called *Sculpting Algorithm* [10, 25], that computes automatically (if it exists) the safe set given a map, a region K in phase space and admissible disturbances and controls. Our goal here is to compute the safe set for the supply based on demand model for the naive case described in the previous section. The Sculpting Algorithm works in such a way that it rejects, in the first iteration, the points *k*_*n*_ for which *k*_*n*+1_ = *f*(*k*_*n*_) + *ξ*_*n*_ need a control *ξ*_*n*_ < *u*_0_ to get back to the region K. The points that survive are a subset of K, and the process is repeated until it finally converges. As a result, we obtain the safe set containing all safe points, which is formed by those points that are controlled with admissible disturbances and controls.

## Controlling the price trajectories in the naive supplier case

Here, we will demonstrate how the naive supplier can prevent a market collapse by only applying the partial control strategy on the selling price. From the naive supplier model shown in Eqs (6–8), we derive the map for the price in Eq (9) to which we will apply the partial control method,

$$\begin{array}{c} D_{n+1}=a-bP_{n+1}, \end{array}$$ (6)

$$\begin{array}{c} S_{n+1}=D_{n}, \end{array}$$ (7)

$$\begin{array}{c} P_{n+1}=\frac{1}{1-M}\cdot \left(\frac{F_{c}}{S_{n+1}}+v-vS_{n+1}+{\left(S_{n+1}\right)}^{2}\right). \end{array}$$ (8)

Our goal is to find the safe set for the following map of the price,

$$\begin{array}{c} P_{n+1}=\frac{1}{1-M}\cdot (\frac{F_{c}}{a-bP_{n}}+v-v\left(a-bP_{n}\right)+{\left(a-bP_{n}\right)}^{2}). \end{array}$$ (9)

We have chosen the parameter values as follow, *M* = 0.5, *F*_*c*_ = 20, *v* = 2, *a* = 10 and *b* = 0.095. These parameter values correspond to the region after the boundary crisis, since we are interested in the transient chaotic regime as we saw in the previous section. In order to apply the Sculpting Algorithm to find the safe set, we need to define a region K in the phase space where the map is acting and where we want the dynamics to stay in. Then, we can compute an admissible choice of disturbances and controls.

**Fig 4:**
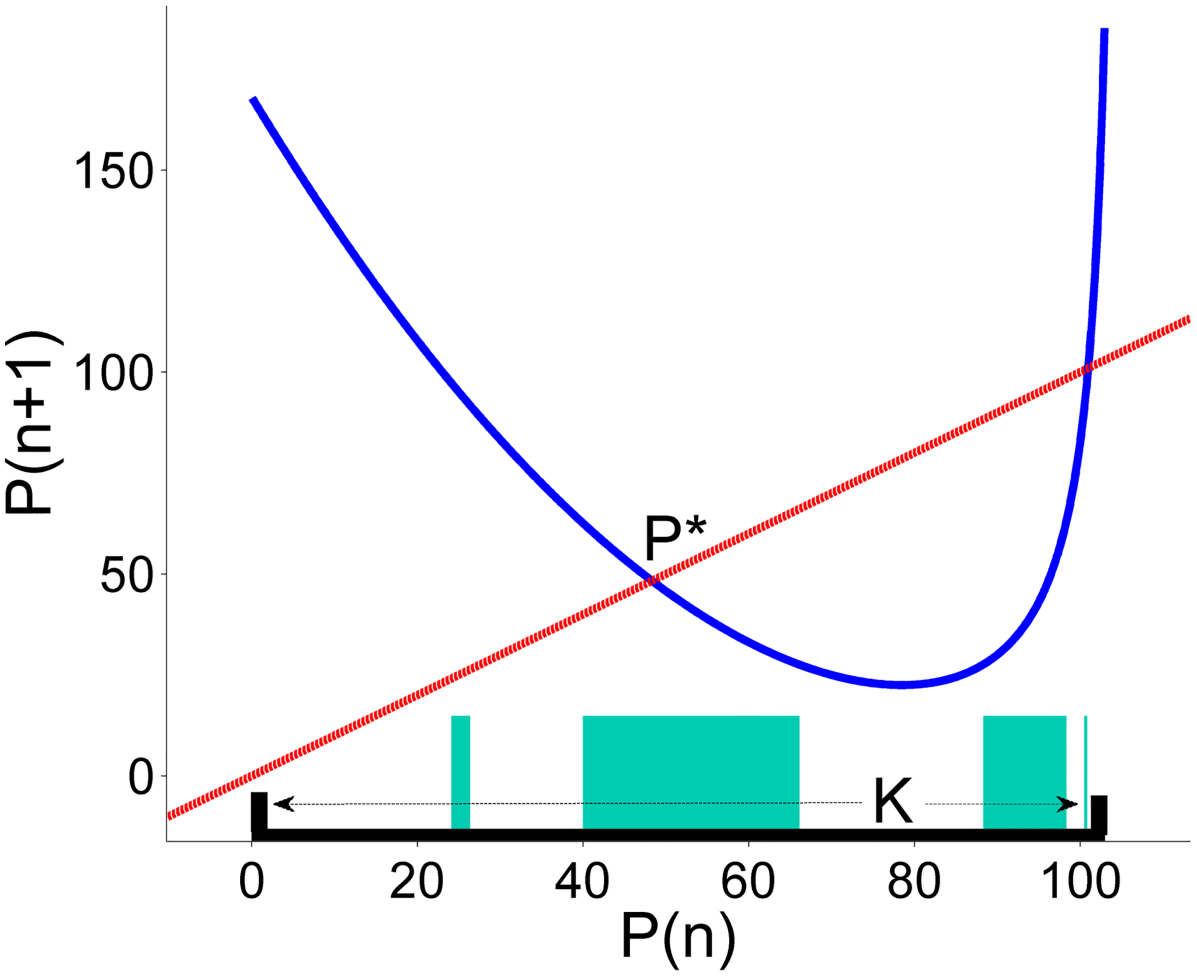
The phase space of the price map. This figure shows the phase space of the price map (blue line) *P*_*n*+1_ = *f*(*P*_*n*_) and the region K (black thick line over the horizontal) where we want to sustain the dynamics. Using the Sculpting algorithm we have computed the safe set that correspond to an admissible choice of disturbances and controls. Then, we have plotted them as turquoise rectangles over the region K.

We know that the iterates of any initial point for which *P*_*n*_ > P*, follow a chaotic dynamics until they finally asymptotes to infinity when they cross the critical value *P*_*n*_ < P*, which actually implies uncontrolled growing price fluctuations, that provoke the market collapse. We have defined the initial region K in the phase space where we want to maintain the dynamics of the system as follows. There is a critical price value P* ≃ 48.838, in which if the supplier prices the product above it, the market will collapse in some close future. The upper bound of the region K is subject to the production function of the supplier, and the lower bound of the region K is assumed to be zero. The critical price value P* ≃ 48.838 must be inside the region K to ensure the success of the control strategy. We have chosen for our simulations the initial region K to be the interval *P*_*n*_ ∈ [0, 103] see Fig 4. Note, that region the K contains the chaotic saddle, which is the responsible for the existence of the chaotic transient.

Furthermore, we have chosen a uniform noise distribution bounded by *ξ*_0_, where a disturbance |*ξ*_*n*_| ≤ *ξ*_0_ is introduced each time step. Other probability distributions are possible in the partial control method, as shown in [29, 30]. However we will only use uniform distributions in this paper for the sake of simplicity. The reader can think of the disturbance as any unpredictable positive or negative change in the price, that was not taken into account in the pricing process. Oil prices may be a good example for that. Consider a situation where the price of oil suddenly goes up, incrementing the transportation costs. This random fluctuation will influence immediately on the selling price. When the firm did not have time to change its margin or the variable costs considering this unpredictable fluctuation, it can influence the price applying some control. The control term in contrast is not random at all. The firm applies it with the only purpose of controlling the price trajectory avoiding the market collapse. We want to remind that the firm controls the price without the intend of maximizing profits, he uses this control method only with the objective of maintaining the “business alive”. The firm makes discounts when the price is high or inflate the price when the price is low, in order to control the long term trajectory of the price. Those ups and downs in the price affect only the gross margin of the supplier. The new margin is easy to compute including the control term.

Now, we can use the Sculpting Algorithm [25] in order to find the safe sets. The computation of the safe set depends on the chosen values of *ξ*_0_ and *u*_0_ and our observations indicate that for a given *ξ*_0_, we may obtain different safe sets which correspond to different values of *u*_0_. The lower the *u*_0_ bound the smaller the final safe set is. Nevertheless, there is a critical value of *u*_0_, below which no safe set exists. We have chosen for our numerical simulations *ξ*_0_ = 10 and *u*_0_ = 6.82, where *u*_0_ is very close to the minimum value for which safe sets exists. When the trajectory is in the region K, we evaluate the value of *f*(*P*_*n*_) + *ξ*_*n*_. If the point is inside the safe set we do not apply the control, and if it is outside, we relocate it inside the nearest safe point, resulting the new safe point *P*_*n*+1_ = *f*(*P*_*n*_) + *ξ*_*n*_ + *u*_*n*_. The final result of this computation gives rise the safe set as shown in Fig 4.

**Fig 5:**
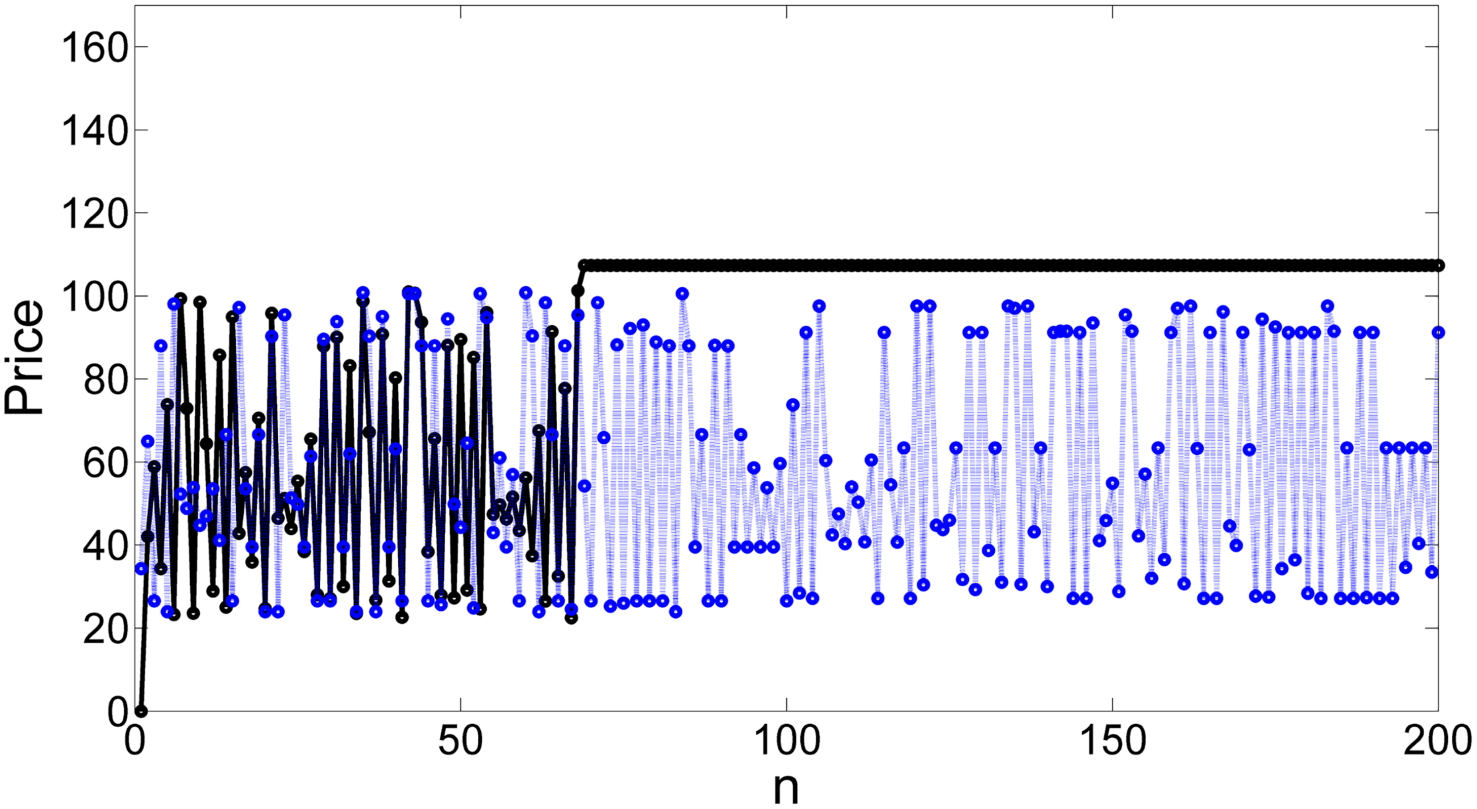
Controlled time series of price. Black line: time series of the price without control exhibiting an escape towards some high price level in which no trade can be done. Blue dot line: controlled time series of the price where the market collapse is avoided. This time series corresponds to the first 200 iterations of the system.

**Fig 6:**
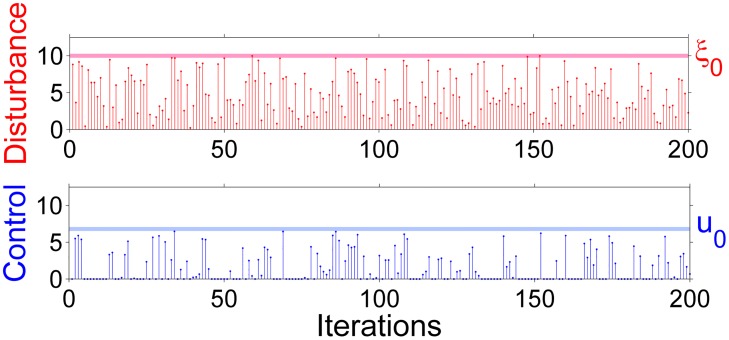
The disturbance and control applied in absolute values at each time step. Note that the control applied in order to sustain the trajectory in the transient regime is always smaller than the disturbance. The reader can check that every time step the amount of control (blue bar) is much smaller than the amount of the disturbance (red bar), where the average control disturbance ratio is 0.66.

The time series displayed in Fig 5 shows clearly how the firm avoids the price in which the trade becomes impossible using the partial control method. The reader can see the controlled trajectory in blue versus the uncontrolled trajectory in black. Furthermore, the amount of control needed at each time step to maintain the dynamics of price in the transient regime is much smaller than the disturbances, as shown in Fig 6.

## Controlling the quantity demanded in the naive supplier case

**Fig 7:**
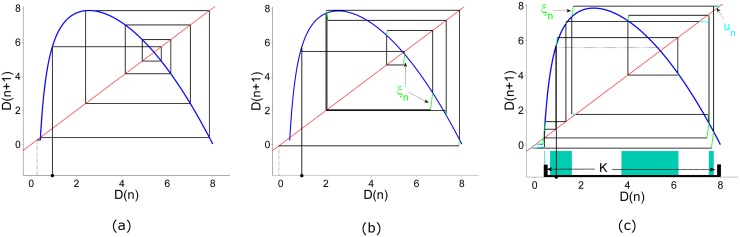
Cobweb plots of the demand uncontrolled and controlled orbit. The blue line represents the demand map, the diagonal red dash line represents *D*_*n*+1_ = *D*_*n*_. The initial condition in all panels is *D*(1) = 0.932. When no disturbances nor control are present in the system, after a few time steps the trajectory escapes from the chaotic saddle (black dotted line) as shown in panel (a). When a disturbance, *ξ*_0_ = 1.0 is present in the system the trajectory escapes from the chaotic saddle even faster as shown in panel (b). The green lines represent the amount of disturbance introduced into the system at each time step. Notice that each line has a different length because it is defined by a uniform distribution function bounded by *ξ*_0_ = 1.0. Panel (c) shows the region K (black thick line over the horizontal) which is the region where we want to sustain the dynamics. Using the Sculpting Algorithm we have found the safe set and we have plotted them as turquoise rectangles over the region K. A control term bounded by *u*_0_ = 0.66 is applied each time step (cyan lines) preventing the collapse.

The demand is much more difficult to control than the price, because it is a variable that depends on other preferences and actions and it is an unaccessible variable to the agent who tries to control it. Moreover, driving the demand is a very expensive task, and it might be done only by the most powerful agents in the economy, such as, large market share companies or the government. We can apply the partial control method in two different conceptual frameworks of our model, the retail market and the wholesale market. In the retail market the firm can influence the demand directly, using a massive advertising and promotional campaigns or even buy its own goods at the market price when there is an excess supply. When a powerful firm is sitting on the demand side of the model representing the entire demand for a much smaller firm, we are modeling a wholesale market situation. Intuitively, these two firms depend completely on each other. The small firm has only one client—the powerful firm. In the same way, the powerful firm has only one supplier—the small firm. The powerful firm can force the small firm to match its production with its own needs, but it must be very careful in not stressing the small firm too much. By stressing here we mean that there are some demand patterns that lead to the collapse of the small firm, which would have a catastrophic effect on the powerful firm. This is a reason important enough for the big firm to control the demand in the system. Technically speaking, it is simple to apply partial control to solve these problems in the context of our model and it can be done in a very efficient way. As in the previous section, we will begin finding the safe sets. We derived the map for the demand in Eq (10) from the naive supplier model presented in Eqs (6–8). We have followed the same strategy as before in order to define the region K. The firm estimates the potential amount of goods that can be consumed in the market *D*_*max*_. As we can see in Fig 7, almost every initial demand *D*_0_ sooner or later is repelled to minus infinity. Thus, no matter what quantity is chosen by the firm for *D*_*max*_, the system will lay on a transient chaos regime. We have chosen for the numerical simulations the initial region K to be the interval *D*_*n*_ ∈ [0.4, 8] as shown in panel (c) in Fig 7. The iterative map for *D*_*n*_ is then as follows,

$$\begin{array}{c} D_{n+1}=a-b(\frac{1}{1-M}\cdot (\frac{F_{c}}{D_{n}}+v-vD_{n}+{\left(D_{n}\right)}^{2})). \end{array}$$ (10)

We have introduced a noise term to the system exactly as in the previous section. This disturbance represents an unexpected demand. For example, an unpredictable new trend or an unpredictable seasonal effect. If the firm sees that a positive control is needed, it might intervene directly in the market, buying the indispensable amount of products that ensure the demand to be met. Massive promotional and advertising campaigns can be used to achieve the same goal in an indirect manner. It is clear that, those two possibilities are very expensive, hence, just powerful firms can afford such expensive interventions in the economy. As in the previous section, there is a critical value of *u*_0_, below which no safe set exists. We have chosen for our numerical simulation *ξ*_0_ = 1.0 and *u*_0_ = 0.66, where *u*_0_ is very close to the minimum value for which safe sets exists. When the trajectory is out of the safe set, we evaluate the value of *f*(*D*_*n*_) + *ξ*_*n*_. If the point is inside a safe set, we do not apply the control, and if it is outside, we relocate it inside the nearest safe point, resulting the new safe point *D*_*n*+1_ = *f*(*D*_*n*_) + *ξ*_*n*_ + *u*_*n*_. The final result of the application of the Sculpting Algorithm is shown in Fig 7.

**Fig 8:**
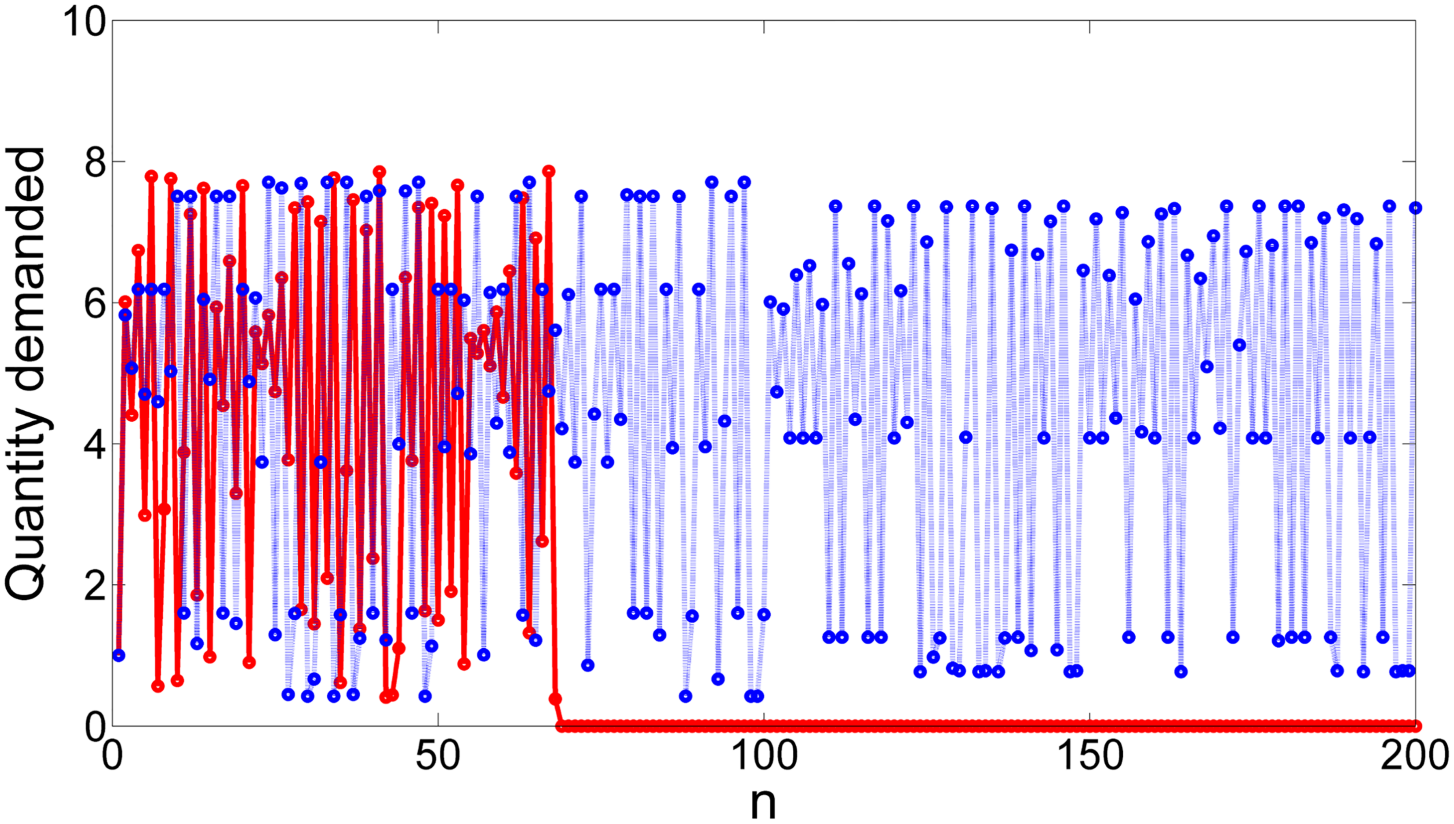
Controlled time series of the quantity demanded. Red line: time series of the quantity demanded without control exhibiting a escape towards zero, what implies an imminent market collapse. Blue dot line: controlled time series of the quantity demanded where the market collapse is avoided. This time series corresponds to 200 iterations of the system.

**Fig 9:**
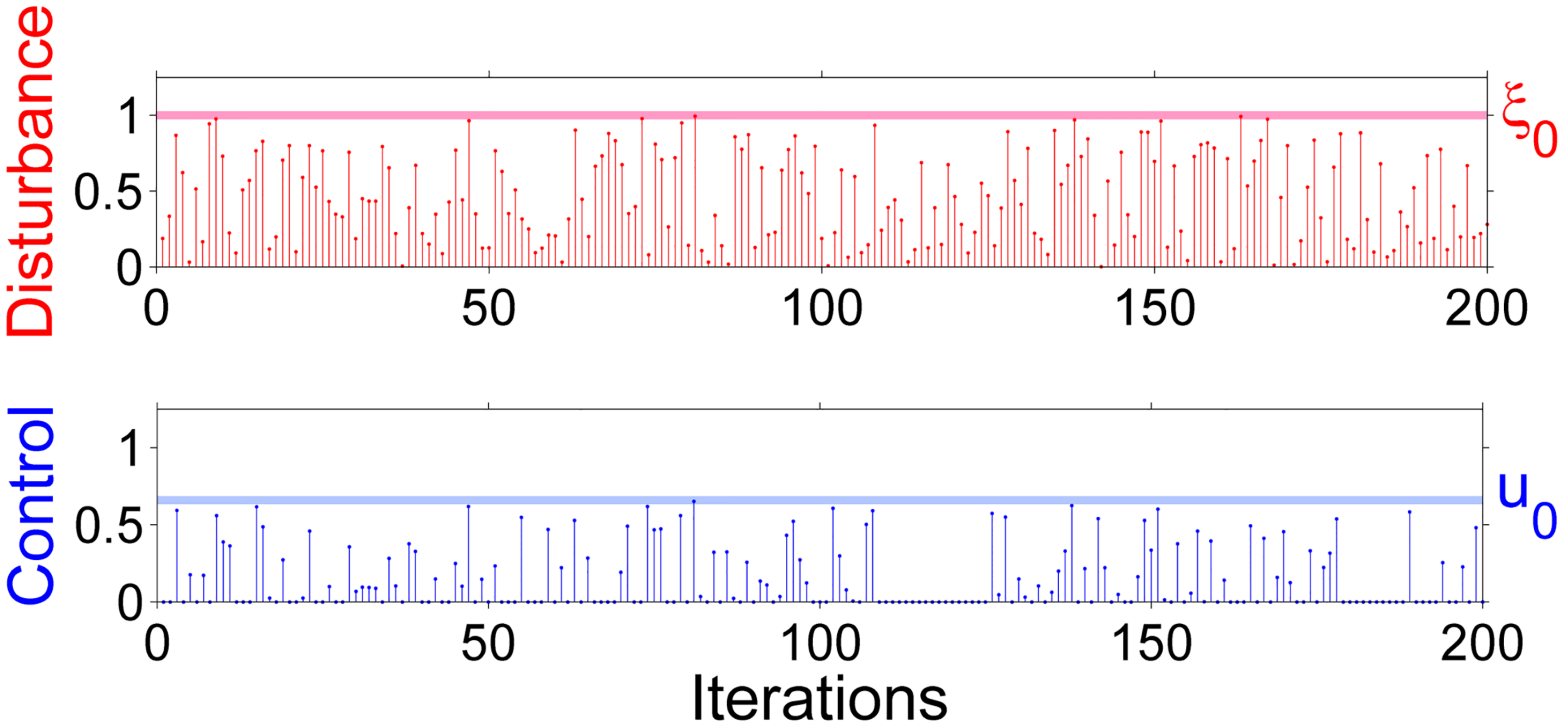
The disturbance and control applied in absolute values at each time step. Note that the control applied in order to sustain the trajectory in the transient regime is always smaller than the disturbance. The reader can check that every time step the amount of control (blue bar) is much smaller than the amount of disturbance (red bar), where the average control disturbance ratio is 0.66 again.

Although in practice there are plenty of difficulties to estimate the demand in the market, the reader can check in the time series in Fig 8 how a powerful firm can actually prevent the demand of crossing the critical point that triggers the disintegration of demand and subsequently the collapse by using the partial control method. Furthermore, the amount of control needed at each time step to maintain the dynamics of demand in the transient regime is much smaller than the disturbances, as shown in Fig 9.

As we mention in the previous sections, the quantity supplied in the time step *n*+1 is simply the quantity demanded at time step *n*. Thinking about this, the supplier may apply the partial control method directly on the quantity supplied.

## Controlling the quantity supplied in the cautious and optimistic supplier case

When the future demand is uncertain, controlling the quantity of supply is a very complex task. In our model we assume a deterministic process where after computing the expected demand the supplier knows exactly the quantity of supply. In this setup, unexpected supply fluctuations are impossible. However, there is a special case where these fluctuations can be considered feasible. In some industrial processes the supplier can only estimate the average quantity of production and not the exact amount. In this context, the partial control method can be used to control the quantity supplied and consequently the market, assuming that the firm always have some extra stock to stream into the market when positive control is needed. To demonstrate the efficiency of the partial control method in complex scenarios where the firm behavior is more sophisticated, we have chosen the cautious and optimistic supplier for this section. The model that represents this type of supplier is shown in Eqs (11–13).

$$\begin{array}{c} D_{n+1}=a-bP_{n+1}, \end{array}$$ (11)

$$\begin{array}{c} S_{n+1}=\sqrt[{}]{\left(\frac{D_{n}}{S_{n}}\right)}\cdot S_{n}, \end{array}$$ (12)

$$\begin{array}{c} P_{n+1}=\frac{1}{1-M}\cdot (\frac{F_{c}}{S_{n+1}}+v-vS_{n+1}+{\left(S_{n+1}\right)}^{2}). \end{array}$$ (13)

**Fig 10:**
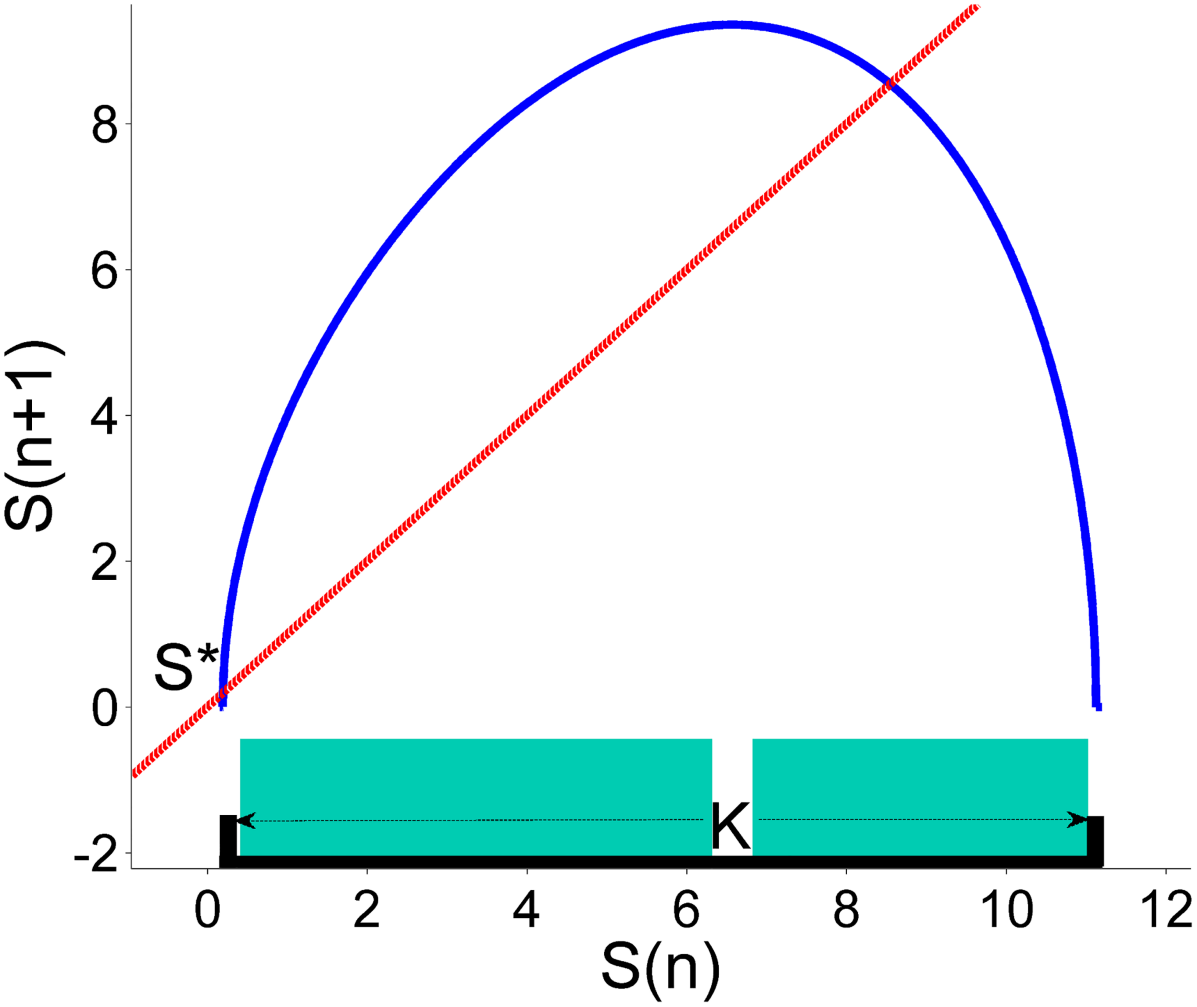
The phase space of the supply map. This figure shows the phase space of the supply map (blue line) and the intersect *S*_*n*+1_ = *S*_*n*_ (red dash line). The region K (black thick line over the horizontal) is the region where we want to sustain the dynamics. Using the Sculpting Algorithm we have found the safe sets and we have plotted them as turquoise rectangles over the region K.

We are interested in the map for the quantity supplied shown in Eq (14) which is a simplification of Eqs (11–13). The region K is defined by the interval between zero and the maximum amount of goods that can be produced using the fix capital *S*_*max*_. Independently to the quantity supplied that is chosen by the firm for *S*_*max*_, the system will almost always lay on a transient chaos regime. We have chosen for our numerical simulations the initial region K to be the interval *S*_*n*_ ∈ [0.15, 11.2] as shown in Fig 10.

$$\begin{array}{c} S_{n+1}=\sqrt[{}]{\frac{1}{S_{n}}(\frac{1}{1-M}\cdot (a-b(\frac{F_{c}}{S_{n}}+v-vS_{n}+S_{n}^{2})))}\times S_{n}. \end{array}$$ (14)

Again, we have introduced a uniform noise distribution bounded by *ξ*_0_. This disturbance can be positive when the quantity produced exceeds the expected production or it can be negative when the quantity produced is below the expected production. When positive control is needed the firm uses its extra stock to fill the shortfall. When negative control is needed the supplier destroys or simply takes out from the market the exceed quantity. As in the previous sections there is a critical value of *u*_0_, below which no safe set exists. We have chosen for our numerical simulation *ξ*_0_ = 2.0 and *u*_0_ = 0.33, where *u*_0_ is very close to the minimum value for which a safe set exists. We evaluate the value of *f*(*S*_*n*_) + *ξ*_*n*_. If the point is inside a safe set, we do not apply the control. If it is outside, we relocate it inside the nearest safe point, resulting the new safe point *S*_*n*+1_ = *f*(*S*_*n*_) + *ξ*_*n*_ + *u*_*n*_. The final result of applying the Sculpting Algorithm is shown in Fig 10.

**Fig 11:**
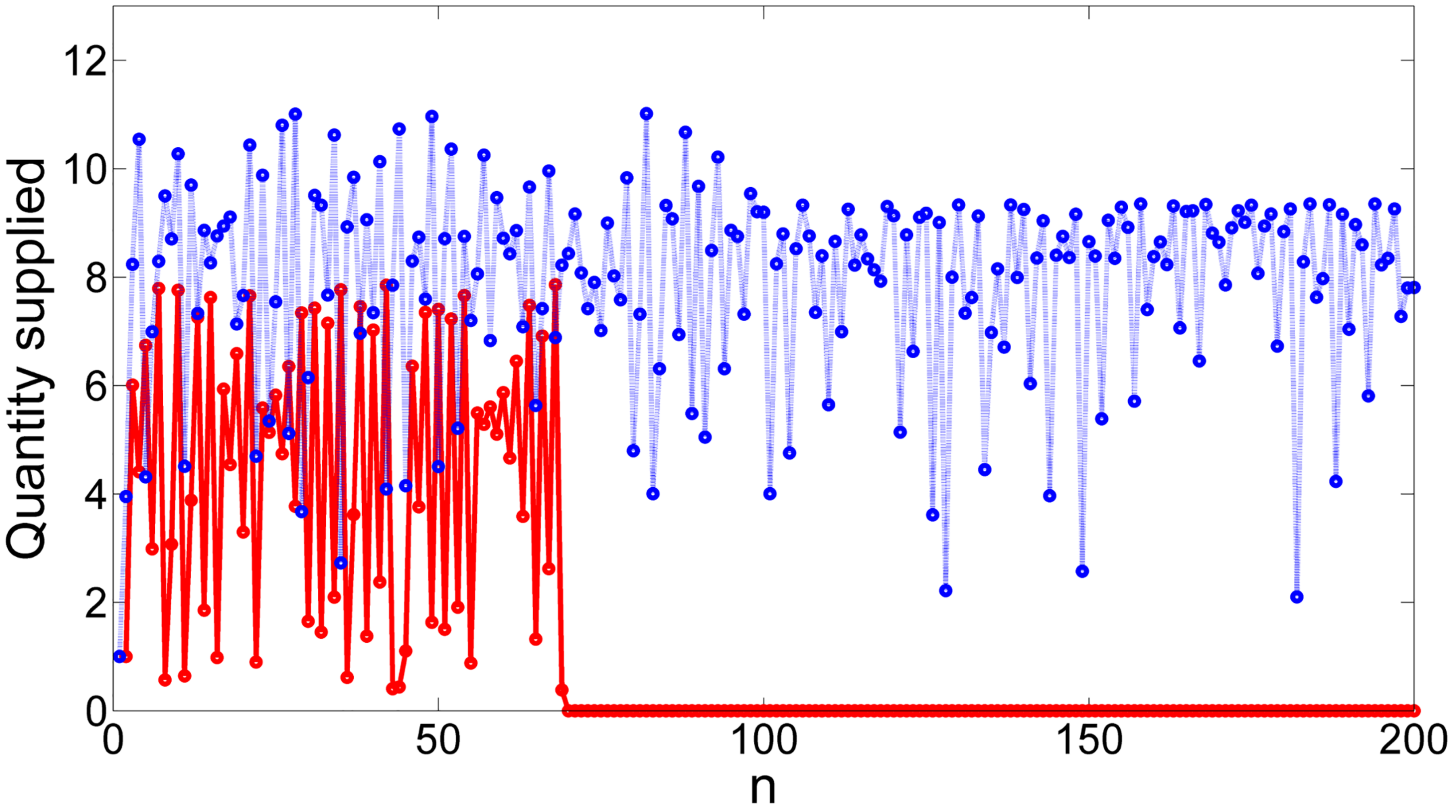
Controlled time series of the quantity supplied. Red line: time series of the quantity supplied without control exhibiting a escape towards zero, what implies an imminent market collapse. Blue dot line: controlled time series of the quantity supplied where the market collapse is avoided. This time series corresponds to 200 iterations of the system.

**Fig 12:**
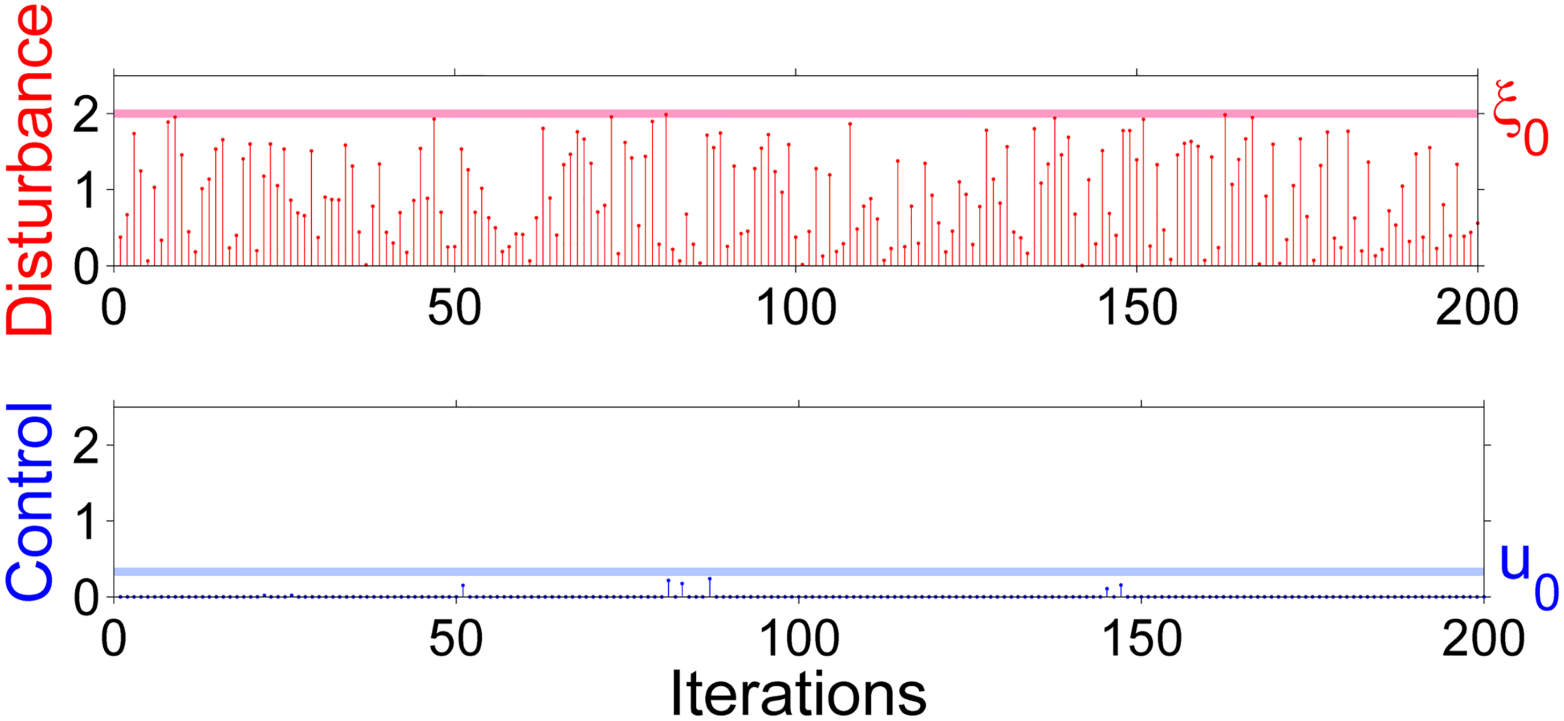
The disturbance and control applied in absolute values at each time step. Note that the control applied in order of sustaining the trajectory in transient regime is always smaller than the disturbance. The control disturbance ratio is much smaller than in the last two sections and is about 0.165.

The efficiency of the partial control method is shown in Fig 11. We want to emphasize another powerful property that can be exploited using the partial control method. The red line in Fig 11, corresponds to the time series of the quantity supplied without control. Originally, the firm can produce a limited amount of products, in this case is around 8 products each time step. We have extended this natural barrier to 11.2 by defining a region K larger then the natural bounds of the system. The controlled trajectory is higher than the uncontrolled trajectory due to the assumption we made earlier, letting the firm to introduce positive controls using its extra stock. This interesting property [31] can be exploited by the firm. Assuming that the firm has no limited stock, he can supply all of it, without collapsing the market. Furthermore, the amount of control needed at each time step to maintain the dynamics of supply in the transient regime is much smaller than the disturbances as shown in Fig 12.

## Conclusions

Avoiding market collapses might be a big challenge for economists. The difficulties in predicting such phenomena due to the nonlinear interaction among the agents at the micro level, makes the engineering of a control strategy at the macro level a very hard task. This can be worst when unpredictable external disturbances are present in the system. In this work, we have shown that this macro state of collapse can be avoided acting on the macro level of the market when a powerful firm had influenced the quantity demanded using the partial control method. A firm with high market power might influence the demand by intervening directly in the market, buying the excess supply, or just by investing in advertising to encourage the consumption in the market. But it is more remarkable how the agents at the micro level have avoided the macro state of collapse by changing their behaviors using the partial control strategy. We have used the supply based on demand model to show how the firm can control the price trajectory, only by changing the selling price of the product at every time step in accordance to the market circumstances. We have also shown that the firm can apply the partial control strategy on the quantity supplied in some special cases. Furthermore, while it is doing that, it is able to extend the natural barriers of the system supplying more goods than before without being detrimental to the market. We have used the partial control method, that has the advantage of using a control to sustain the dynamics in the transient regime much smaller than the external disturbances introduced in the model. The pursuit for efficient control strategies to help humans dominate the economy has always been there. The wounds left by the last global crisis are a painful reminder of why we need to insist in this search. Novel control methods like the partial control method bring us closer to realize this dream.

## References

[1] In V, Spano ML, Neff JD, Ditto WL, Daw CS, Edwards KD, et al Maintenance of chaos in a computational model of a thermal pulse combustor. Chaos. 1997;7:606–613. 10.1063/1.166260 12779686

[2] Dangoisse D, Glorieux P, Hannequin D. Laser chaotic attractors in crisis. Phys Rev Lett. 1986;57:2657–2660. 10.1103/PhysRevLett.57.2657 10033827

[3] Dhamala M, Lai YC. Controlling transient chaos in deterministic flows with applications to electrical power systems and ecology. Phys Rev E. 1999;59:1646–1655. 10.1103/PhysRevE.59.1646

[4] Duarte J, Januário C, Martins N, Sardanyés J. Chaos and crises in a model for cooperative hunting: a symbolic dynamics approach. Chaos. 2009;58:863–883. 10.1063/1.3243924 20059198

[5] Chiarella C, Dieci R, Gardini L. Speculative behaviour and complex asset price dynamics: a global analysis. J Econ Behav Organ. 2002;49:173–197. 10.1016/S0167-2681(02)00066-5

[6] Day RH, Huang W. Bulls, bears and market sheep. J Econ Behav Organ. 1990;14:299–329. 10.1016/0167-2681(90)90061-H

[7] Tramontana F, Gardini L, Dieci R, Westerhoff F. The emergence of “bull and bear” dynamics in a nonlinear model of interacting markets. Discrete Dyn Nat Soc. 2009;2009:310471 10.1155/2009/310471

[8] Chian AC-L, Rempel EL, Rogers C. Complex economic dynamics: Chaotic saddle, crisis and intermittency. Chaos Solitons Fractals. 2006;29:1194–1218. 10.1016/j.chaos.2005.08.218

[9] Wu W, Chen Z, Ip WH. Complex nonlinear dynamics and controlling chaos in a Cournot duopoly economic model. Nonlinear Anal Real World Appl. 2010;11:4363–4377. 10.1016/j.nonrwa.2010.05.022

[10] Sabuco J, Zambrano S, Sanjuán MAF. Partial control of chaotic systems using escape times. New J Phys. 2010;12:113038 10.1088/1367-2630/12/11/113038

[11] Levi A, Sabuco J, Sanjuán MAF. Supply based on demand dynamical model; 2017. Preprint. Available from: arXiv:1701.07333.

[12] Ezekiel M. The cobweb theorem. Q J Econ. 1938;52:255–280. 10.2307/1881734

[13] Hommes CH. Dynamics of cobweb model with adaptive expectations and nonlinear supply and demand. J Econ Behav Organ. 1994;24:315–335. 10.1016/0167-2681(94)90039-6

[14] Petruzzi NC, Maqbool D. Pricing and the Newsvendor Problem: A Review with Extensions. Oper Res. 1999;47(2):183–194. 10.1287/opre.47.2.183

[15] Sornette D. Why Stock Markets Crash: Critical Events in Complex Financial Systems Princeton: Princeton University Press; 2003.

[16] Sornette D. Dragon-Kings, Black Swans and the Prediction of Crises. Int J Terraspace Sci Eng. 2009;2(1):1–18

[17] Sornette D, Johansen A, 2001. Significance of log-periodic precursors to financial crashes. Quant Finance. 2001;1(4):452–471. 10.1088/1469-7688/1/4/305

[18] Harmon D, De Aguiar MAM, Chinellato DD, Braha D, Epstein IR, Bar-Yam Y. Predicting economic market crises using measures of collective panic; 2011. Preprint. Available from: arXiv:1102.2620v1. Cited 28 July 2017.

[19] Ladislav k. Fractal Markets Hypothesis and the Global Financial Crisis: Scaling, Investment Horizons and Liquidity; 2012. Preprint. Available from: arXiv:1203.4979v1. Cited 32 July 2017.

[20] Ladislav k. Fractal Markets Hypothesis and the Global Financial Crisis: Wavelet Power Evidence. Sci Rep. 2013;3:2857 10.1038/srep02857 24091386 PMC3790199

[21] Janusz AH, Tilo H, Giinter H, Wolfgang W. How to control a chaotic economy? J Evol Econ. 1996;6:31–42. 10.1007/BF01202371

[22] Zambrano S, Sanjuán MAF, Yorke JA. Partial control of chaotic systems. Phys Rev E. 2008;77:055201(R). 10.1103/PhysRevE.77.055201 18643119

[23] Zambrano S, Sanjuán MAF. Exploring partial control of chaotic systems. Phys Rev E. 2009;79:026217 10.1103/PhysRevE.79.026217 19391830

[24] Sabuco J, Sanjuán MAF, Yorke JA. Dynamics of partial control. Chaos. 2012;22:047507 10.1063/1.4754874 23278093

[25] Sabuco J, Zambrano S, Sanjuán MAF, Yorke JA. Finding safety in partially controllable chaotic systems. Commun Nonlinear Sci Numer Simul. 2012;17:4274–4280. 10.1016/j.cnsns.2012.02.033

[26] Batten D. Discovering Artificial Economics: How agents learn and economies evolve. Boulder: Westview Press; 2000.

[27] Zambrano S, Sabuco J, Sanjuán MAF. How to minimize the control frequency to sustain transient chaos using partial control. Commun Nonlinear Sci Numer Simul. 2014;19:726–737. 10.1016/j.cnsns.2013.06.016

[28] Capeáns R, Sabuco J, Sanjuán MAF, Yorke JA. Partially controlling transient chaos in the Lorenz equations. Philos Trans R Soc Lond A. 2017;375:20160439. 10.1098/rsta.2016.0211 PMC5311432 28115608

[29] Coccolo M., Seoane J., Zambrano S., Sanjuán MAF. Partial Control of Escapes in Chaotic Scatering. Int J Bifurcat Chaos. 2013;23:1350008 10.1142/S0218127413500089

[30] Agarwal V., Sabuco J., Balachandran B. Safe regions with partial control of a chaotic system in the presence of white Gaussian noise. Int J Nonlin Mech. 2017 10.1016/j.ijnonlinmec.2017.01.017

[31] Das S, Yorke JA. Avoiding extremes using partial control. J Differ Equ Appl. 2016;22:217–234. 10.1080/10236198.2015.1079181

